# Recurrent/Subsequent Stroke and Associated Outcomes in Geriatric Patients with OSA and Prior Stroke Events: A Retrospective Study Using the 2019 National Inpatient Sample

**DOI:** 10.3390/jpm13050782

**Published:** 2023-04-30

**Authors:** Rupak Desai, Sandeep Singh, Sai Priyanka Mellacheruvu, Adil Sarvar Mohammed, Roshni Soni, Ayodya Perera, Venkata Akhil Makarla, Sarayu Santhosh, Muneeb Ali Siddiqui, Bilal Khan Mohammed, Zaki Ur Rahman Mohammed, Zainab Gandhi, Ankit Vyas, Akhil Jain, Rajesh Sachdeva, Gautam Kumar

**Affiliations:** 1 Division of Cardiology, Atlanta VA Medical Center, 1670 Clairmont Road, Decatur, GA 30033, USA; 2 Department of Internal Medicine, University Hospital of North Midlands NHS Foundation Trust, Stoke-on-Trent ST4 6QG, UK; 3 Department of Public Health, University of Massachusetts, Lowell, MA 01854, USA; 4 Department of Clinical Research, Mayo Clinic, Phoenix, AZ 85054, USA; 5 Department of General Medicine, GMERS Medical College, Gotri, Vadodara 390021, India; 6 International Faculty of Medicine, Tbilisi State Medical University, Tbilisi 0186, Georgia; 7 Department of Internal Medicine, Mamata Medical College, Khammam 507002, India; 8 Department of Internal Medicine, Adichunchanagiri Institute of Medical Sciences, Rajiv Gandhi University of Health Sciences, B.G. Nagara, Bengaluru 560041, India; 9 College of Osteopathic Medicine, William Carey University, Hattiesburg, MS 39401, USA; 10 Department of Clinical Research, Duke University Medical Center, Durham, NC 27710, USA; 11 Department of Internal Medicine, Sanford Health, Fargo, ND 58102, USA; 12 Department of Internal Medicine, Wyoming Valley Medical Center, Wilkes-Barre, PA 18711, USA; 13 Department of Internal Medicine, Baptist Hospitals Of Southeast Texas, Beaumont, TX 77701, USA; 14 Department of Internal Medicine, Mercy Catholic Medical Center, Darby, PA 19153, USA; 15 Division of Cardiology, Emory University School of Medicine, Atlanta VA Medical Center, Atlanta, GA 30322, USA

**Keywords:** obstructive sleep apnea, stroke/transient ischemic attack, subsequent/recurrent stroke, previous history of stroke, mortality, predictors, disparities

## Abstract

**Background**: Obstructive sleep apnea (OSA) increases the risk of stroke and cardiovascular diseases. However, its impact on geriatric patients with a prior history of stroke/transient ischemic attack (TIA) has not been adequately studied. **Methods:** We utilized the 2019 National Inpatient Sample in the US to identify geriatric patients with OSA (G-OSA) who had a prior history of stroke/TIA. We then compared subsequent stroke (SS) rates among sex and race subgroups. We also compared the demographics and comorbidities of SS+ and SS− groups and utilized logistic regression models to assess outcomes. **Results:** Out of 133,545 G-OSA patients admitted with a prior history of stroke/TIA, 4.9% (6520) had SS. Males had a higher prevalence of SS, while Asian-Pacific Islanders and Native Americans had the highest prevalence of SS, followed by Whites, Blacks, and Hispanics. The SS+ group had higher all-cause in-hospital mortality rates, with Hispanics showing the highest rate compared to Whites and Blacks (10.6% vs. 4.9% vs. 4.4%, *p* < 0.001), respectively. Adjusted analysis for covariates showed that complicated and uncomplicated hypertension (aOR 2.17 [95% CI 1.78–2.64]; 3.18 [95% CI 2.58–3.92]), diabetes with chronic complications (aOR 1.28 [95% CI 1.08–1.51]), hyperlipidemia (aOR 1.24 [95% CI 1.08–1.43]), and thyroid disorders (aOR 1.69 [95% CI 1.14–2.49]) were independent predictors of SS. The SS+ group had fewer routine discharges and higher healthcare costs. **Conclusions:** Our study shows that about 5% of G-OSA patients with a prior history of stroke/TIA are at risk of hospitalization due to SS, which is associated with higher mortality and healthcare utilization. Complicated and uncomplicated hypertension, diabetes with chronic complications, hyperlipidemia, thyroid disorders, and admission to rural hospitals predict subsequent stroke.

## 1. Introduction

Obstructive Sleep Apnea (OSA) has been recognized as a risk factor for stroke and cardiovascular events, and the prevalence of OSA is particularly high in elderly patients [1]. A prior stroke or transient ischemic attack (TIA) is also associated with an increased risk of subsequent/recurrent stroke [2]. However, little is known about the burden and impact of OSA on recurrent/subsequent stroke and associated hospitalization outcomes in geriatric patients (G-OSA) with prior stroke/TIA. The current study aims to investigate the predictors of subsequent/recurrent stroke and associated sex/racial disparities and in-hospital outcomes in elderly patients with OSA and prior stroke/TIA.

Several studies have investigated the relationship between OSA and stroke in the general population [3,4]. A systematic review and meta-analysis showed that sleep disturbance is associated with an increased risk of recurrent cardiovascular and cerebrovascular events in patients who have already experienced a stroke [1]. In addition, screening for OSA in stroke patients may identify those at risk for stroke recurrence [2]. However, there is a lack of information about the relationship between OSA and subsequent/recurrent stroke in geriatric patients with prior stroke/TIA. Moreover, little is known about the impact of sex and race on this relationship.

The current study addresses these knowledge gaps using data from the National Inpatient Sample (2019). We will compare the rate of subsequent stroke by sex and racial subgroups and assess the prevalence of subsequent in-hospital all-cause mortality in G-OSA patients with prior stroke/TIA. We also identified independent predictors of subsequent stroke in this patient population. The findings from this study will provide important insights into the burden, predictors, and impact of recurrent/subsequent stroke and associated in-hospital outcomes in elderly patients with OSA and prior stroke/TIA.

## 2. Methods

### 2.1. Data Source

We used the National Inpatient Sample (NIS) (2019), a publicly available database that is part of the Healthcare Cost and Utilization Project (HCUP) sponsored by the Agency for Healthcare Research and Quality (AHRQ) [5] and contains information from over 35 million hospitalizations each year from non-federal acute care hospitals in 45 states of the United States as our data source.

### 2.2. Study Population

**Figure 1:**
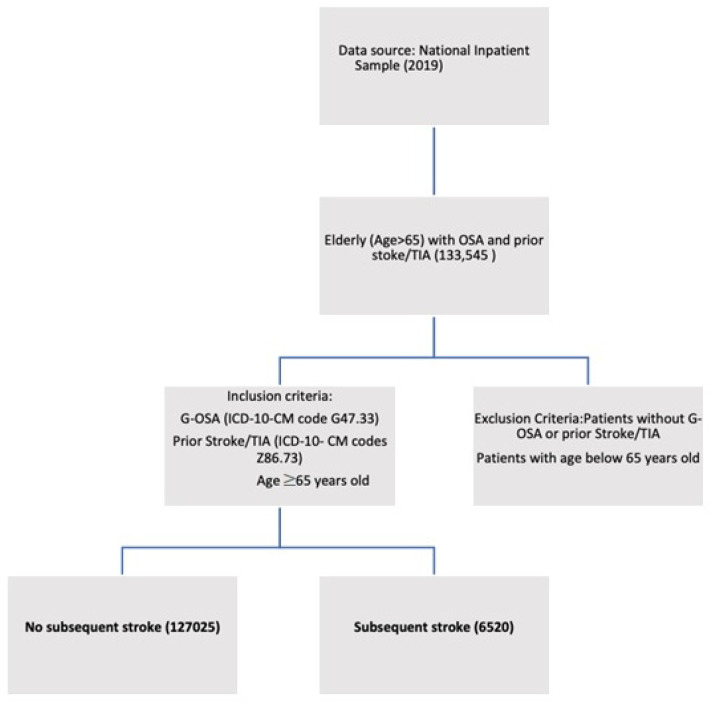
Flowchart describing study protocol.

Elderly patients aged 65 or older with OSA and prior stroke/transient ischemic stroke (TIA) were identified using the NIS (2019). Patients with a diagnosis of G-OSA (ICD-10-CM code G47.33) and prior stroke/TIA (ICD-10-CM codes Z86.73) were included in the study. We excluded patients with missing data on age, sex, race, and comorbidities. Weighted data were used to calculate the national estimates of hospitalizations for the study population. Figure 1 describes the study population selection protocol.

### 2.3. Statistical Analysis

We compared the rate of subsequent stroke (SS) in gender and racial subgroups. We further subdivided the G-OSA patients with and without SS (SS+ vs. SS−) and compared the demographics and comorbidities between the two groups. We used multivariable logistic regression models to identify independent predictors of SS in G-OSA patients with prior stroke/TIA, adjusted for age, sex, race, and comorbidities like hypertension, diabetes, dyslipidemia, coronary artery disease, congestive heart failure, atrial fibrillation, chronic kidney disease, and smoking. The primary outcomes of this study included the prevalence of SS, subsequent in-hospital all-cause mortality, and multivariate predictors of SS in G-OSA patients with prior stroke/TIA. Secondary outcomes included healthcare resource utilization between the SS+ vs. SS− cohorts.

We used IBM SPSS Statistics version 25.0 (IBM Corp., Armonk, NY, USA) with complex survey modules to perform all analyses. We used the Pearson chi-square test for categorical variables and the Mann-Whitney U test for continuous variables based on the non-normal distribution of the data. A *p*-value of less than 0.05 was considered statistically significant. The cell sizes of less than 11 were suppressed per the HCUP privacy guidelines [6]. Ethical approval was not required for this study as the NIS database does not contain any identifiable information about patients.

## 3. Results

A total of 133,545 elderly patients with OSA (G-OSA) and prior stroke or TIA were included in this study. From these, an age-matched SS+ (6520) cohort and SS− (127,025) cohort were identified. Among all the elderly patients (4.9%) had a SS during the hospitalization, including 3.7% with acute ischemic stroke (AIS). The mean age of both groups was 75 years (Interquartile range of 70–81). On subgroup analysis based on racial and gender distribution, the rate of SS was highest in Asian/Pacific Islanders (6%) and Native Americans (5.6%), followed by whites, blacks, and Hispanics (4.9%, 4.6%, and 4.1%, respectively). Males had a higher prevalence of SS than females (5.2% vs. 4.4%; *p* < 0.001). 

Among the cardiovascular comorbidities, patients in the SS+ cohort were more likely to have uncomplicated hypertension (42.7% vs. 29.7%), hyperlipidemia (76.5% vs. 71.7%), and the SS- cohort was more likely to have prior MI (17.6% vs. 14.1%), and prior percutaneous coronary intervention (1.4% vs. 0.8%) (all *p* < 0.001) [Table 1]. Among the endocrinal comorbidities, the rates of diabetes without chronic complications (17.9% vs. 12.8%) were higher in the SS+ group, and the rates of obesity (35.3% vs. 29.9%) were high in the SS− group. Psychiatric-related disorders such as alcohol use (2.8% vs. 1.6%) were more prevalent among the SS+ cohort, and depression (20.7% vs. 17.7%) was more prevalent in the SS− cohort (all *p* < 0.001). Furthermore, the all-cause in-hospital mortality was higher in the SS+ cohort (5.1% vs. 1.6%; *p* < 0.001), which was particularly high in Hispanics compared to whites and blacks (10.6% vs. 4.9% vs. 4.4%; *p* < 0.001), with no gender disparity noticed.

As shown in Table 2, the multivariate logistic regression analysis revealed that several factors were associated with subsequent stroke in elderly patients with prior OSA and prior stroke/TIA. Non-elective admission was strongly associated with subsequent stroke, with a six-fold increased odds ratio compared to elective admission (OR 6.506, 95%CI 4.832–8.760 *p* < 0.001). Significant clinical predictors of SS in G-OSA patients on multivariable regression analysis were complicated and uncomplicated hypertension (aOR 2.17 [95% CI 1.78–2.64]; 3.18 [2.58–3.92]); diabetes with chronic complications (aOR 1.28 [95% CI 1.08–1.51]); hyperlipidemia (aOR 1.24 [95% CI 1.08–1.43]); and thyroid disorders (aOR 1.69 [95% CI 1.14–2.49]). However, patients with obesity (aOR 0.86 [0.76–0.98]), prior myocardial infarction (aOR 1.27 [95% CI 1.22–1.33]), and chronic pulmonary disease (aOR 0.54 [95% CI 0.47–0.62]) had lower odds of SS. The SS+ cohort had fewer routine discharges and higher healthcare costs than the SS− cohort.

In summary, this study demonstrated that nearly 5% of G-OSA patients with prior stroke/TIA history were hospitalized with subsequent stroke, associated with a significantly higher mortality rate and healthcare resource utilization. Comorbidities, including hypertension, diabetes, dyslipidemia, and admissions in rural hospitals, were independent predictors of having SS and required preventive measures for secondary prevention of stroke. The study also identified sex and racial disparities in the burden of subsequent stroke in G-OSA patients with prior stroke/TIA.

## 4. Discussion

Obstructive sleep apnea (OSA) is a common sleep disorder that affects millions of people worldwide, particularly elderly individuals. Previous studies established that OSA is associated with an increased risk of stroke and other cardiovascular events. However, the burden of subsequent or recurrent stroke (SS) and its predictors in geriatric patients with OSA who had a prior history of stroke or transient ischemic attack (TIA) remains poorly understood. This study aimed to address this knowledge gap by analyzing data from the National Inpatient Sample of 2019.

The study revealed that 4.9% of geriatric patients with OSA and prior stroke/TIA had SS, including 3.7% acute ischemic stroke (AIS). The study also found that male and Asian/Pacific Islander patients had a higher risk of SS than females and other racial subgroups, respectively. Another significant finding in our study was that patients with SS had a significantly higher mortality rate and increased healthcare resource use than those without SS. Factors such as hypertension, diabetes, dyslipidemia, other thyroid disorders, and admission to rural hospitals were independent predictors of subsequent stroke, highlighting the importance of proactive measures for secondary stroke prevention. These findings are consistent with previous studies showing the association between these comorbidities and an increased risk of stroke and cardiovascular disease [7,8]. The increased risk of subsequent stroke observed in our study could be attributed to the underlying pathophysiology of OSA, such as intermittent hypoxia, sympathetic activation, and endothelial dysfunction, which can exacerbate pre-existing cerebrovascular disease and increase the risk of recurrent stroke [9,10]. In a study conducted by Leino et al., OSA polygraphic phenotypes in stroke and TIA patients were compared to reference patients. OSA phenotypes differ in stroke and TIA patients [11]. Shorter episodes have been linked to higher all-cause mortality, but it is unclear if polygraphic traits are present before stroke or altered by acute stroke treatment [11]. A low arousal threshold, light, and fragmented sleep may explain the brief event durations. The study emphasizes the importance of routine OSA screening in acute cerebrovascular illness patients, even without a prior suspicion [11]. Stroke and TIA patients have a high prevalence of OSA, which can worsen stroke rehabilitation outcomes and increase the risk of recurrent cardiovascular events [11]. The Berlin Questionnaire (BQ), Epworth Sleepiness Scale (ESS), STOP-BANG, and SACS did not significantly predict OSA on formal polysomnography (PSG) in patients with post-ischemic stroke or transient TIA [12]. Sico et al. developed and validated a cerebrovascular disease-specific OSA prediction model to identify patients at risk for OSA and compare its performance to commonly used screening instruments in patients with ischemic stroke and transient ischemic attack [12]. A clinical prediction model based on patient symptomatology and routinely gathered demographic, anthropometric, medical history, and stroke severity data could, however, be applied to this group [12]. OSA was observed in many post-stroke or TIA patients, underscoring the importance of identifying OSA risk factors and vascular risk factors [12].

Interestingly our study showed a negative association between obesity and subsequent stroke, contrary to previous studies [7,13] that have consistently shown obesity to be a risk factor for stroke. This may be due to the study population being limited to elderly patients with prior OSA and prior stroke/TIA, who may have different risk factors for subsequent stroke than the general population. Our study also showed a higher all-cause in-hospital mortality in the SS+ cohort compared to the SS− cohort. This finding is consistent with previous studies demonstrating an increased mortality risk in patients with OSA and stroke [9,14]. Moreover, the higher mortality rate observed in Hispanics compared to other racial groups may be attributed to disparities in access to care, socioeconomic factors, and cultural barriers [15,16]. Therefore, efforts should be made to improve access to care and provide culturally appropriate care to improve outcomes in this vulnerable population.

These findings have several important clinical implications. First, elderly patients with OSA and prior stroke/TIA, particularly male and Asian/Pacific Islander patients, should be closely monitored for the development of subsequent strokes. Patients with OSA are typically not screened for OSA in busy stroke clinics and inpatient settings, as the stroke team is typically overburdened with work and must address higher-priority health issues [2]. This may prevent many stroke patients from missing the screening for OSA, leading to subsequent strokes [2]. Consequently, the role of Sleep Educators can enhance screening [2]. The sleep educator is a sleep center nurse with training in sleep medicine. This increases the diagnosis and treatment of OSA without placing an undue burden on the stroke team [2]. Our study’s racial and gender subgroup analysis highlights the need for targeted interventions in certain high-risk populations to reduce the incidence of SS. These findings are crucial for clinicians and policymakers to develop effective prevention strategies for secondary stroke and cardiovascular disease in geriatric patients with OSA. Second, comorbidities, including hypertension, diabetes, and hyperlipidemia, should be identified and treated promptly in these patients, as they were identified as independent predictors of SS. Third, interventions aimed at reducing the burden of OSA in elderly patients with prior stroke/TIA, such as continuous positive airway pressure (CPAP) therapy, may be beneficial in reducing the risk of subsequent strokes and improving in-hospital outcomes [17,18]. It is essential to acknowledge the importance of early-age screening in preventing OSA and the consequent risk of major adverse cardiac and cerebrovascular events [19]. Nasal surgery improves hypoventilation, nasal resistance, and compliance with CPAP therapy in OSA patients but is often overlooked in patient management [20]. Nasal septal deviation or turbinal hypertrophy does not represent a site of upper airway collapse, but nasal surgery has been shown to improve CPAP compliance and snoring severity [19].

The study has important limitations that should be taken into account. First, the retrospective study design and use of administrative data limit our ability to account for potential confounding factors. Second, due to the lack of a control group, we were unable to investigate whether or not OSA severity was a predictor of recurrent stroke. However, our study focused on identifying predictors of in-hospital outcomes in geriatric OSA patients with prior stroke events. Third, due to the constraints of our study’s retrospective design and the data source employed (2019 National Inpatient Sample), we could not gather information on OSA severity, such as Apnea-Hypopnea Index (AHI) and treatment status, from the dataset. Furthermore, the applicability of our findings to outpatients may have been constrained by the fact that our study only included hospitalized patients. The study, which may be a key indicator of SS in older patients with OSA and a history of stroke or TIA, failed to investigate the connection between the severity of OSA and the risk of future strokes. The study did not evaluate how CPAP therapy affected the risk of future strokes in elderly people with OSA and a history of stroke or TIA, to name just a few of the limitations. Future research should examine these limitations and potential risk factors for a second stroke in older adults with OSA and a history of a stroke or TIA.

## 5. Conclusions

The present study provides compelling evidence that the burden of OSA in geriatric patients with prior stroke or TIA is associated with a higher risk of SS and all-cause in-hospital mortality. Our findings reveal that 4.9% of G-OSA patients with a prior history of stroke/TIA were hospitalized with SS, with a higher prevalence in Asian/Pacific Islanders and Native Americans and in males compared to females. The study’s findings underscore the need for close monitoring and prompt treatment of comorbidities, including hypertension, diabetes, and dyslipidemia, in these patients and suggest that interventions aimed at reducing the burden of OSA may be beneficial in improving in-hospital outcomes and reducing the risk of subsequent strokes. Future studies should focus on assessing the impact of OSA severity and CPAP therapy on the risk of subsequent strokes in this population to inform clinical practice and improve patient outcomes and explore novel strategies for secondary prevention of stroke in G-OSA patients with a focus on optimizing management of modifiable risk factors.

## Figures and Tables

**Table 1:** Baseline characteristics of hospitalized elderly patients with prior OSA and prior stroke/TIA.

Variables		No Subsequent Stroke (*n* = 127,025)	Subsequent Stroke (*n* = 6520)		
		Percentage %	Percentage %		*p*-Value
Age (years) at admission	Median [IQR]	75 (70–81)	75 (70–81)		<0.001
Sex	Male	57.40%	61.40%		<0.001
	Female	42.60%	38.60%		
Race	White	81.20%	82.00%		<0.001
	Black	11.50%	10.70%		
	Hispanic	4.40%	3.70%		
	Asian/Pacific Islanders	1.10%	1.50%		
	Native Americans	0.50%	0.50%		
	Others	1.30%	1.60%		
Median household income national quartile for patient ZIP Code	0–25th	25.90%	26.50%		<0.001
	26–50th	27.00%	25.10%		
	51–75th	26.30%	25.40%		
	76–100th	20.80%	23.00%		
Primary expected payer	Medicare	90.10%	89.00%		0.003
	Medicaid	0.60%	0.80%		
	Private including HMO	6.80%	7.70%		
	Self-pay	0.40%	0.20%		
	No charges	0.00%	0.00%		
	Others	2.10%	2.20%		
Type of admission	Non-elective	81.30%	95.30%		<0.001
	Elective	18.70%	4.70%		
Bed size of the hospital	Small	21.00%	16.50%		<0.001
	Medium	28.10%	29.80%		
	Large	50.90%	53.70%		
Location/teaching status of the hospital	Rural	8.40%	5.80%		<0.001
	Urban non-teaching	17.80%	16.70%		
	Urban teaching	73.80%	77.50%		
Region of hospital	North-East	14.20%	13.40%		<0.001
	Mid-West	33.80%	30.70%		
	South	36.10%	36.00%		
	West	15.90%	19.90%		
**Comorbidities**					
Acquired immune deficiency syndrome		0.20%	0.20%		0.817
Alcohol abuse		1.60%	2.80%		<0.001
Arthropathies		5.90%	3.80%		<0.001
Leukemia		0.80%	0.70%		0.288
Lymphoma		1.00%	0.60%		0.001
Metastatic cancer		1.80%	2.10%		0.109
Solid tumor without metastasis, in situ		0.00%	0.20%		<0.001
Solid tumor without metastasis, malignant		3.20%	2.90%		0.266
Dementia		12.50%	12.50%		0.93
Depression		20.70%	17.70%		<0.001
Hypertension, complicated		50.40%	48.10%		<0.001
Hypertension, uncomplicated		29.70%	42.70%		<0.001
Diabetes with chronic complications		40.20%	34.20%		<0.001
Diabetes without chronic complications		12.80%	17.90%		<0.001
Hyperlipidemia		71.70%	76.50%		<0.001
Obesity		35.30%	29.90%		<0.001
Peripheral vascular disease		13.20%	13.00%		0.637
Prior MI		17.60%	14.10%		<0.001
Prior PCI		1.40%	0.80%		<0.001
Prior CABG		17.00%	14.90%		<0.001
Tobacco Use Disorder		7.40%	8.20%		0.019
Chronic pulmonary disease		42.40%	27.70%		<0.001
Hypothyroidism		22.30%	20.50%		<0.001
Other thyroid disorders		1.40%	2.40%		<0.001
Cancer		6.60%	6.20%		0.174
**In-hospital outcomes**					
All-cause mortality		1.60%	5.10%		<0.001
All-cause mortality by gender		**Male**	**Female**		
	**No stroke**	1.80%	1.30%		<0.001
	**Stroke**	5.20%	4.80%		0.407
All-cause mortality by race		**White**	**Black**	**Hispanic**	
	**No stroke**	1.60%	1.50%	1.60%	0.088
	**Stroke**	4.90%	4.40%	10.60%	<0.001
Disposition of patient	Routine	46.20%	32.30%		<0.001
	Transfers to Short term hospital	1.90%	2.80%		
	Transfer–other: Includes Skilled Nursing Facility (SNF), Intermediate Care Facility (ICF), Another type of facility	25.50%	42.20%		
	Home Health Care (HHC)	24.40%	17.30%		
	Against Medical Advice (AMA)	0.40%	0.30%		
Length of stay (days)	Median [IQR]	4	3		<0.001
Total charges (USD)	Median [IQR]	$39,035	$42,865		<0.001

OSA = obstructive sleep apnea; TIA = transient ischemic attack; IQR = Interquartile range; PCI = Percutaneous coronary intervention; MI = Myocardial infarction; CABG = Coronary artery bypass grafting; other thyroid disorders. Other thyroid disorders were identified using the Elixhauser comorbidity tool using these ICD-10 CM codes, E040, E041, E042, E048, E049, E0500, E0501, E0510, E0511, E0520, E0521, E0530, E0531, E0540, E0541, E0580, E0581, E0590, E0591, E060, E061, E062, E063, E064, E065, E069, O905. *p* < 0.05 was considered statistically significant.

**Table 2:** Multivariate logistic regression for predictors of subsequent stroke in elderly patients with OSA and prior stroke/TIA.

Variables	Adjusted Odds Ratio	95% CI		*p* Value
		Lower	Upper	
Female	1.11	0.98	1.25	0.096
Non-elective vs. Elective admission	6.51	4.83	8.76	<0.001
**Location/teaching status of the hospital**				
Urban non-teaching vs. Rural	1.48	1.09	2.03	0.008
Urban teaching vs. Rural	1.68	1.27	2.23	
**Comorbidities**				
Hypertension complicated	2.17	1.78	2.64	<0.001
Hypertension Uncomplicated	3.18	2.58	3.92	<0.001
Diabetes with chronic complications	1.28	1.08	1.51	0.004
Hyperlipidemia	1.24	1.08	1.43	0.002
Obesity	0.86	0.76	0.98	0.025
Prior myocardial infarction	0.8	0.67	0.96	0.016
Chronic pulmonary disease	0.54	0.47	0.62	<0.001
Other thyroid disorders	1.69	1.14	2.49	0.009

OSA = obstructive sleep apnea; TIA = transient ischemic attack; CI = Confidence Interval; other thyroid disorders = Abnormality of thyroid-binding globulin, Hemorrhage of thyroid, and Infarction of thyroid. Multivariable regression analysis was adjusted for age, sex, race, and cardiovascular and extra-cardiovascular comorbidities. *p* < 0.05 was considered statistically significant.

## Data Availability

The data utilized in this research are available from the author upon request. The information is not available to the public because of privacy restrictions.
